# Phenotypic and genotypic characterization of *Staphylococci* causing breast peri-implant infections in oncologic patients

**DOI:** 10.1186/s12866-015-0368-x

**Published:** 2015-02-10

**Authors:** Ramona Barbieri, Marianna Pesce, Simonetta Franchelli, Ilaria Baldelli, Andrea De Maria, Anna Marchese

**Affiliations:** Microbiology Unit DISC, University of Genoa, Genoa, Italy; IRCCS-AOU San Martino IST, Reconstruction Plastic Surgery Unit, DISC, University of Genoa, Genoa, Italy; IRCCS-AOU San Martino IST, Reconstruction Plastic Surgery Unit, Genoa, Italy; IRCCS-AOU San Martino IST, Infectious Diseases Unit, DISSAL, University of Genoa, Genoa, Italy; IRCCS-AOU San Martino IST, Microbiology Unit DISC, University of Genoa, Largo Rosanna Benzi 10, 16132 Genoa, Italy

**Keywords:** Implant infections, Biofilm, Staphylococcal infections, *ica* gene, biofilm-related genes, MSCRAMMs

## Abstract

**Background:**

*Staphylococcus epidermidis* and *S. aureus* have been identified as the most common bacteria responsible for sub-clinical and overt breast implant infections and their ability to form biofilm on the implant as been reported as the essential factor in the development of this type of infections. Biofilm formation is a complex process with the participation of several distinct molecules, whose relative importance in different clinical settings has not yet been fully elucidated. To our knowledge this is the first study aimed at characterizing isolates causing breast peri-implant infections.

**Results:**

Thirteen *S. aureus* and seven *S. epidermidis* causing breast peri-implant infections were studied.

Using the broth microdilution method and the E-test, the majority of the strains were susceptible to all antibiotics tested. Methicillin resistance was detected in two *S. epidermidis*. All strains had different RAPD profiles and were able to produce biofilms in microtitre plate assays but, while all *S. aureus* carried and were able to express *icaA* and *icaD* genes, this was only true for one *S. epidermidis.* Biofilm development was glucose- and NaCl-induced (5 *S. aureus* and 1 *S. epidermidis*) or glucose-induced (the remaining strains). Proteinase K and sodium metaperiodate treatment had different effects on biofilms dispersion revealing that the strains studied were able to produce chemically different types of extracellular matrix mediating biofilm formation.

All *S. aureus* strains harboured and expressed the *atlA, clfA, FnA, eno* and *cna* genes and the majority also carried and expressed the *sasG* (10/13), *ebpS* (10/13) genes.

All *S. epidermidis* strains harboured and expressed the *atlE*, *aae, embp* genes, and the majority (six strains) also carried and expressed the *fbe, aap* genes.

Genes for *S. aureus* capsular types 5 and 8 were almost equally distributed. The only leukotoxin genes detected were l*uk*E/l*uk*D (6/13).

**Conclusions:**

*S. aureus* and *S. epidermidis* breast peri-implant infections are caused by heterogeneous strains with different biofilm development mechanisms.

Since the collagen adhesin (*cna)* gene is not ubiquitously distributed among *S. aureus*, this protein could have an important role in the cause of breast peri-implant infections.

**Electronic supplementary material:**

The online version of this article (doi:10.1186/s12866-015-0368-x) contains supplementary material, which is available to authorized users.

## Background

Immediate breast reconstruction, using tissue expanders and implants, has become a standard of care for almost all women requiring mastectomy for breast cancer [1-3]. However, after implantation, patients may experience local complications during the ensuing years, with peri-implant infection being one of the most common problems, with an infection rate ranging from 0 to 29 percent [4].

The most traditional approach to severe or refractory breast periprosthetic infection remains removal of the device. However, removal makes subsequent reinsertion and re-expansion more difficult, since the soft tissue retracts rapidly to close the expanded pocket [5].

The majority of cases reported identify *Staphylococcus epidermidis* and *S. aureus* as the most common bacteria responsible for sub-clinical and overt breast implant infections.

Bacteria may reach the implants during or after placement and their ability to form biofilms on the implant has been reported as the essential factor in the development and persistence of infection. Biofilm formation is a complex process with the participation of several distinct molecules, whose relative importance in different clinical settings has not yet been fully elucidated.

Specific staphylococcal surface proteins impacting adhesion to abiotic surfaces (AtlA, AtlE, Bap/Bhp) as well as a vast array of proteins called MSCRAMMs (Microbial Surface Components Recognizing Adhesive Matrix Molecules) promoting colonization of medical implants have been identified [6]. In *S. aureus* and *S. epidermidis*, production of an extracellular polysaccharide promoting intercellular adhesion (PIA), also called polymeric N-acetyl-glucosamine (PNAG) is currently the best-characterised biofilm mechanism [7]. PIA/PNAG is synthesised by enzymes encoded by the ica operon.

Once this operon is activated, four proteins are transcribed *ica*A, *ica* D, *ica*B and *ica*C. The expression of *ica*A alone induces low enzymatic activity, however, the simultaneous expression of *ica*A and *ica*D promotes a significant increase in the amount of polysaccharide [8].

However, recent studies have begun to highlight the existence of PIA/PNAG-independent biofilm mechanisms in both species [9]. Accumulation-associated protein (Aap) independently or toghether with *ica* operon, has also been suggested to be important in coagulase-negative staphylococci [10]. In *S. aureus* and *S. epidermidis* more additional surface proteins such as SasG, extracellular matrix binding protein (Embp), biofilm-associated protein (Bap) and the fibronectin-binding proteins FnbpA and B, were implicated in matrix formation. These findings suggest that other surface proteins may also be involved in biofilm development. In some strains, biofilm formation may be mediated additionally or exclusively by specific surface proteins (Bap/Bhp and Aap) [6].

While several studies have extensively described the distribution of genes involved in biofilm formation and virulence in Staphylococcal strains causing orthopaedic peri-implant infections, to our knowledge, this is the first study characterizing isolates causing breast peri-implant infections.

## Results

### Antimicrobial susceptibility and *mecA* presence

All *S. aureus* isolates were susceptible to, oxacillin, co-trimoxazole, daptomycin, gentamicin, linezolid, rifampin, tetracycline and vancomycin. Ciprofloxacin- and erythromycin resistance was detected in one (1/13, 7.7%) and three (3/13, 23.1%) strains, respectively (Table 1). Two out of the three erythromycin-resistant *S. aureus* showed inducible clindamycin resistance.

**Table 1:** **Susceptibility of 20 Staphylococci isolated from breast implant infections**

		**Range**	**MIC50**	**MIC90**	**%S**
*S. aureus* (13)	ciprofloxacin	≤0.12-4	≤0.12	1	92.3
	cotrimoxazole	≤2/38	≤2/38	≤2/38	100
	clindamycin	≤0.25	≤0.25	≤0.25	100*
	erythromycin	≤0.25- ≥ 8	≤0.25	≥8	76.9
	tetracycline	≤1	≤1	≤1	100
	rifampicin	≤0.5	≤0.5	≤0.5	100
	linezolid	1	1	1	100
	vancomycin	≤0.5-1	1	1	100
	oxacillin	≤0.25-1	0.5	1	100
	gentamicin	≤0.25-0.5	0.5	0.5	100
	daptomycin	0.12-1	0.25	1	100
*S. epidermidis* (7)	ciprofloxacin	≤0.12			100
	cotrimoxazole	≤2/38-4			85.7
	clindamycin	≤0.25			100
	erythromycin	≤0.25- ≥ 8			28.6
	tetracycline	≤1			100
	rifampicin	≤0.5			100
	linezolid	1			100
	vancomycin	1			100
	oxacillin	≤0.25-4			71.4
	gentamicin	≤0.25- > 16			71.4
	daptomycin	0.25-0.5			100

*Two strains showed inducible clindamycin resistance.

All *S. epidermidis* were susceptible to, ciprofloxacin, clindamycin, daptomycin, linezolid, rifampin, tetracycline and vancomycin. Oxacillin, erythromycin, gentamicin and co-trimoxazole resistance was detected in 2/7, 5/7, 2/7 and 1/7 strains, respectively.

The oxacillin susceptibility results were confirmed by cefoxitin disk test and *mecA* PCR**.**

### Biofilm assays

All *S. aureus* isolates*,* and only one out of seven *S. epidermidis* isolates were classified as slime producers by the Congo Red Agar (CRA) plate method, developing brown-black (*S. aureus*) or black (*S. epidermidis*) colonies.

All isolates were classified as biofilm-producing strains by the polystyrene microtiter plates (MtP) method.

**Figure 1:**
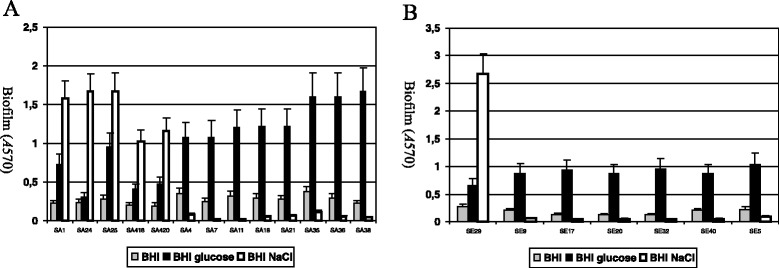
**Biofilm formation in 13** ***S. aureus*** **(A) and 7** ***S. epidermidis*** **(B) strains studied.** The strains were grown in BHI medium or in BHI supplemented with 1% glucose or 4% NaCl at 37°C for 24 h. Biofilm formation in tissue culture-treated 96-well plates was measured three times, and standard deviations, which were less than 20%, are indicated.

When grown in brain heart infusion (BHI) supplemented with 1% glucose, biofilm development was significantly induced in twelve *S. aureus* and six *S. epidermidis* strains while 4% NaCl activated biofilm development in five *S. aureus* and one *S. epidermidis* strain (Figure 1). For these six strains NaCl was a stronger biofilm inducer than glucose.

**Figure 2:**
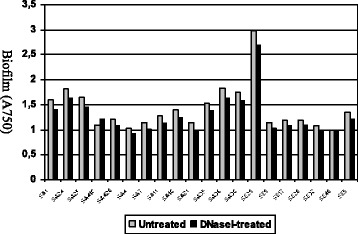
**Susceptibility of S.** ***aureus*** **and** ***S. epidermidis*** **biofilms to treatment with DNase I.** Strains SA 1, SA 24, SA 25, SA 418, SA 420 and SE 29 were grown in BHI supplemented with 4% NaCl at 37°C for 24 h prior to treatment. The remaining strains were grown in BHI supplemented with 1% glucose at 37°C for 24 h prior to treatment.

**Figure 3:**
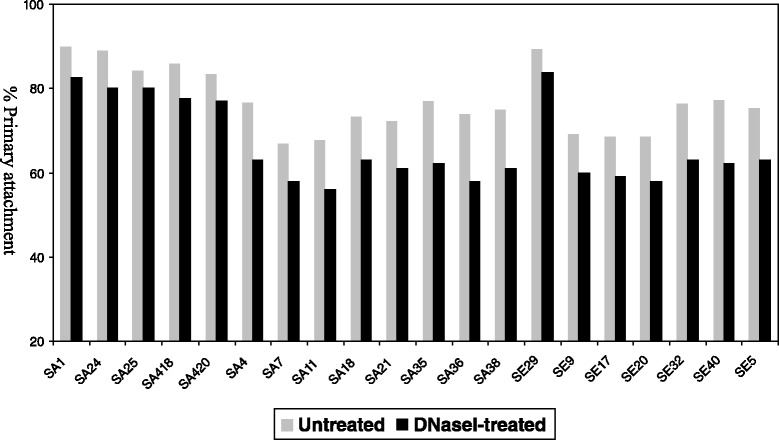
**Effect of DNase I on** ***S. aureus*** **and** ***S. epidermidis*** **primary attachment.** Strains SA 1, SA 24, SA 25, SA 418, SA 420 and SE 29 were grown in BHI supplemented with 4% NaCl at 37°C for 24 h prior to treatment. The remaining strains were grown in BHI supplemented with 1% glucose at 37°C for 24 h prior to dilution and treatment.

Addition of DNase I did not affect bacterial biofilm stability. Although not statistically significant the lowest percentages of reduction of primary attachment (<10%) were observed for those strains producing NaCl inducible biofilms (Figures 2 and 3).

**Figure 4:**
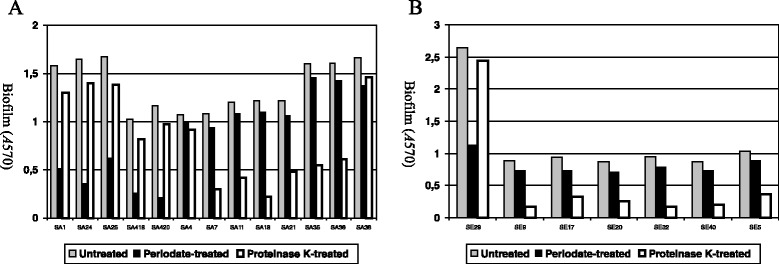
**Susceptibility of** ***S. aureus*** **(A) and** ***S. epidermidis*** **(B) biofilms to treatment with sodium metaperiodate and proteinase K.** Strains SA 1, SA 24, SA 25, SA 418, SA 420 and SE 29 were grown in BHI supplemented with 4% NaCl at 37°C for 24 h prior to treatment. The remaining strains were grown in BHI supplemented with 1% glucose at 37°C for 24 h prior to treatment.

All NaCl-induced biofilms were susceptibile to sodium metaperiodate (>70% reduction in optical density (OD)) and resistant to proteinase *K* (Figure 4). All the remaining biofilms (significantly or only slightly glucose-induced) were resistant to sodium metaperiodate and substantially dispersed by treatment with proteinase K (>60% reduction in OD), with the exception of those produced by two *S. aureus* strains (both proteinase K and sodium metaperiodate resistant).

The different behaviours observed suggested different chemical compositions in the biofilm extracellular matrices.

### Detection of MSCRAMM and biofilm genes

All *S. aureus* harboured the *icaA/D, atlA, clfA, FnA, eno* and *cna* genes and the great majority carried *sasG* (10/13), *ebpS* (10/13) and *fib* (9/13), while only two strains carried *FnB* (Table 2). No strain harboured the *bap* and *bbp* genes.

**Table 2:** **Distribution of MSCRAMM and biofilm genes among** ***S. aureus*** **and** ***S. epidermidis***

	***ebpS***	***eno***	***AtlA/E***	***aae***	***aap***	***bap/bhp***	***sasG***	***cna***	***bbp***	***fnbA***	***fnbB***	***clfA***	***clfB***	***fbe***	***sdrF***	***embp***	***fib***	***icaA/D***
*S. aureus* (13)	10	13	13	n. t.	n.t.	0	10	13	0	13	2	13	8	n.t	n.t	n.t	9	13
*S. epidermidis* (7)	n.t.	n.t.	7	7	6	0	n.t.	n.t.	n.t.	0	0	n.t.	n.t.	6	4	7	n.t.	2*

*1 strain was *icaA* negative and *icaD* positive.

Two *S. epidermidis* carried *ica* genes. One *S. epidermidis* (classified as a slime producer according to the CRA plate method) carried both the *icaA* and *icaD* genes and another strain (classified as a non slime producer, by CRA plate method) was *icaD* positive*.*

All *S. epidermidis* harboured the *atlE*, *aae, embp* genes, the *aap* and the *fbe* genes were carried by six strains, while four strains carried the *SdrF* gene (Table 2). No strains harboured the *bhp* gene (Additional files 1 and 2).

### Expression of MSCRAMM and biofilm genes

Under the experimental conditions used all strains expressed *icaA*/D genes with the exception of one *S. epidermidis* strain which did not express the *icaD* gene (Additional file 3).

For all *S. aureus* strains the RT-PCR amplicons showed positive expression signals for all the remaining genes tested with the exception of *fib.* All *S. epidermidis* strains expressed the *atlE*, *aae, embp* genes, and six strains out of seven expressed the *fbe, aap* genes.

### Detection of *S. aureus* capsular and leukotoxin genes

Six strains were PCR-positive for capsular type 8 and seven for capsular type 5 genes (Additional file 1).

Seven out of thirteen *S. aureus* strains carried the LukE/LukD leukotoxin genes, while the PVL and LukM leukotoxins genes were not detected.

### Randomly amplified polymorphic DNA (RAPD) polymerase chain reaction (PCR) profiles

To rule out the possibility of infections due to one epidemic *S. aureus* or *S. epidermidis* strain, all isolates (13 *S. aureus* and 7 *S. epidermidis*) underwent RAPD PCR with three and two different primers respectively. All *S. aureus* and all *S. epidermidis* had different RAPD profiles (Additional files 4 and 5).

### *S. aureus* MLST and eBURST analysis

As shown in Table 3, a total of 8 sequence types (STs) were revealed, which were further grouped into 6 clonal complexes (CCs).

**Table 3:** **Clonal relatedness of 13** ***S. aureus*** **isolates and their agr groups**

**Strains**	**agr group**	**Sequence type**	Clonal complex*
**SA1**	I	45	45
**SA4**	III	30	30
**SA7**	I	45	45
**SA 11**	II	15	5
**SA18**	II	1162	10
**SA21**	I	20	20
**SA24**	II	5	5
**SA 25**	I	22	22
**SA35**	II	194	5
**SA 36**	II	5	5
**SA 38**	I	45	45
**SA 418**	I	22	22
**SA 420**	I	22	22

*CCs as defined by eBurst analysis with a stringent group definition with six of seven loci.

### *S. aureus agr* typing

The distribution of *agr* alleles among the 13 *S. aureus* strains is provided in Table 3.

The *agr* type I was predominant (7/13; 53,8%), followed by *agr* type II (5/13; 38,5%). Only one *S. aureus* belonged to *agr* type III.

## Discussion

Staphylococcal infection represents a major concern when associated with breast reconstruction, as it may necessitate additional hospitalization, antibiotic treatment, and in more serious cases removal of the device making subsequent reinsertion and re-expansion more difficult [5].

Theoretically, both endogenous and hospital-acquired staphylococci may gain access to breast implants during or following placement. Our data (susceptible antibiotypes, heterogeneity of the STs and the RAPD profiles) supports a community origin of the infecting microorganisms, in most cases. After accession to surgical implants, the essential factor in the evolution and persistence of infections is the formation of bacterial biofilm around implanted devices [11]. All isolates studied were able to form biofilm on polystyrene surfaces but, in contrast to *S. aureus*, the great majority of *S. epidermidis* isolates did not carry the *ica*A or *ica*D genes. Our results are in agreement with previous studies showing that the *ica*-operon is present and expressed in almost all *S. aureus* isolates, although the absence of *ica*-operon activity does not necessarily impact on the ability of *S. epidermidis* to cause clinical biofilm disease. In this study, only one out of seven *S. epidermidis* strains carried both *icaA* and *icaD*. In a recent letter, Persichetti and colleagues [12], described 10 *S. epidermidis* causing periprosthetic breast infections and capsular contracture, yet they reported only three *ica*A and *ica*D positive strains. Although the number of cases analysed in both studies is limited, it seems that *ica*-independent biofilm formation plays a major role compared to *ica*-dependent mechanisms in *S. epidermidis* breast peri-implant infections. In contrast, all *S. epidermidis* studied carried and expressed the gene for Aap protein, a protein that has been shown to mediate biofilm formation in strains lacking the *ica* genes. Biofilm induction by glucose or NaCl (a known activator of ica operon transcription) had different effects on our strains and revealed that our strains had different mechanisms of biofilm development, irrespective of *ica* operon carriage. Proteinase K and sodium metaperiodate treatment confirmed that strains causing breast peri-implant infection can produce different types of biofilm extracellular matrix (involving polysaccharides, or proteins or both). Although not statistically significant DNase I appeared less effective in reducing bacterial attachment in those strains producing NaCl-induced biofilm, suggesting a minor role of extracellular DNA in these isolates. Our data adds further evidence that, in the context of peri-implant infections, biofilm formation is a complex, phenomenon involving distinct molecules, whose relative importance may vary depending on the different clinical settings. Addition of DNase I did not affect bacterial biofilm stability as already reported [13].

Genes coding for biofilm-associated molecules have been thoroughly studied [14] in *S. aureus* and *S. epidermidis* isolated from prosthetic joint infections (PJI).

The most striking difference observed between *S. aureus* strains isolated from PJI and breast implant infections was the rate of surface adhesin (*sasG*) and collagen adhesin (*cna*) genes found. The isolation of a cna-positive *S. aureus* strain was more probable in isolates from breast implant infections than from PJI (100% *vs* 22-29%) indicating that *cna* is of major importance in the specific context of breast implant infections. Other Authors [15-17] have shown the prevalence of the *cna* gene to be much higher in methicillin-resistant *S. aureus (*MRSA) (93%) than methicillin-sensible *S. aureus (*MSSA) clones (46.6%) and that *cna* positive strains appeared to be associated with capsular type 8. However, our strains were all methicillin sensible (MS) and 7 out of 13 strains carried the *cps5* gene for capsular type 5 and the remaining *cps8*, suggesting that the site of infection may have been the driving force in selecting a subpopulation of *cna* positive *S. aureus* strains irrespective of other characteristics such as methicillin resistance and capsular type.

An elevated breast collagen concentration has been shown to be one of the greatest risk factors for developing breast carcinoma [18].

Breast implants, especially in oncologic patients, due to the increased collagen deposition, could be easily covered by a more dense collagen matrix and thus become more prone to adhesion of *S. aureus* strains able to bind collagen.

In contrast to *S. aureus* causing PJI, the great majority of our strains carried and expressed the *sasG* gene (a gene coding for a surface protein that has been shown to promote biofilm formation and adherence to nasal epithelial cells).

This difference in distribution according to the site of infection, is remarkable as its *S. epidermidis* homologue *aap* (accumulation-associated protein) appears to be almost ubiquitously present. Further studies are needed to better characterize this protein, its function/s and to understand its role in different types of infections.

The gene coding for Bap, a cell wall protein found in bovine mastitis *S. aureus* isolates, involved in intercellular adhesion and biofilm formation was absent in all *S. aureus* isolates studied. Our results support the suggestion of other authors, that the role of Bap in human infections is doubtful [14].

Besides the ability to form biofilms, other authors have investigated the incidence of genes coding for leukocidal toxins in *S. aureus* isolated from implant orthopaedic infections. Panton-Valentine, LukE/LukD and LukM leukocidins are known to be associated with necrotic skin and soft tissue infections and could promote damage to peri-implant tissues. *lukE/lukD* was the only gene found in strains from breast implant infections (53.8%). Leukotoxin genes displayed a similar prevalence in strains from breast implants and PJIs, suggesting that LukE/LukD could be the only relevant leukocidin in both types of implant-related infections.

The great majority of *S. epidermidis* analysed carried *AtlE, aap*, *aae, embp* and *fbe* genes, a condition already described for *S. epidermidis* isolated from catheter-related bloodstream infections and PJIs as well as for commensal *S. epidermidis*, indicating that these proteins are valuable during both infection and colonization.

In contrast to *S. aureus* only three S. *epidermidis* strains carried a gene (*SdrF)* coding for a surface protein binding collagen. This is not surprising, since adhesins that recognize host proteins coating the device seem to be the primary mechanism of adhesion in *S. aureus*, while *S. epidermidis* initial adherence is probably multifactorial [19].

Breast reconstruction has become a standard of care for almost all women requiring mastectomy for breast cancer. Reduction of mutilation greatly improves the psychological status of women without interfering with oncological treatment, However, the reconstructive approach may be compromised by early or late implant infection in 8% of patients in prospective series [20]. Further studies are needed to confirm our observation and to verify if patients colonized by *cna* positive strains are at higher risk of breast peri-implant infections than those colonized by *cna* negative *S. aureus*. If this holds true, screening of patients before and after reconstructive surgery at regular intervals could be extremely useful to identify those colonized and thus concentrate management, and resources. Should colonization with *cna*-positive strains confirm to be predictive for higher risk of implant infection, effective prophylactic strategies/treatments could be devised to reduce the risk of infection of reconstruction breast implants.

## Conclusions

*S. aureus* and *S. epidermidis* breast peri-implant infections are caused by heterogeneous strains with different biofilm development mechanisms. Since the collagen adhesin (*cna)* gene is not ubiquitously distributed among *S. aureus*, this protein could have an important role in the cause of breast peri-implant infections. Further studies are needed to confirm our observation and eventually recommend screening of patients before and after reconstructive surgery to identify those colonized by *cna*-positive *S. aureus* and at higher risk of peri-implant infections.

## Methods

Twenty staphylococci (13 *S. aureus* isolates and 7 *S. epidermidis* isolates) were studied.

The isolates represented non repetitive strains causing periprosthetic infection in patients who underwent breast implantation (silicone mammary prosthesis) after mastectomy for breast cancer at the Oncology Institute (IST) of Genoa, Italy, between January 2011 and January 2013.

Clinical data and isolates were taken as part of standard patient care and according to this condition the study was exempt from specific ethics commitee scrutiny and approval. Informed consent for the use of clinical data has been obtained by patients.

Infection was defined as a complication occurring after breast implant surgery that was characterized by three or more of the following findings: pain, local swelling, erythema, pus, fever, seroma, wound dehiescence, or perforation of the skin, as previously described [20].

All isolates were identified using a commercial biochemical tests (API Staph identification strip; bioMérieux, Marcy-l’Etoile, France).

Strains were isolated from implants (8), peri-implant fluids (6), capsular tissues (4), a tissue expander (1) and a pus sample (1).

A sonication procedure was adopted to detach adherent bacteria from the expander, implants and capsular tissues [21].

### Susceptibility tests

The minimal inhibitory concentrations (MICs) of oxacillin, ciprofloxacin, gentamicin, erythromycin, clindamycin, co-trimoxazole, tetracycline, rifampin, vancomycin and linezolid were determined by the broth microdilution method following the European committee for Antimicrobial Susceptibility testing (EUCAST) (Version 4.0, 2014) guidelines and interpretative criteria [22,23]. Daptomycin-MICs were determined by E-test (bioMérieux, Marcy-l’Etoile, France) on Muller-Hinton agar (Biolife, Milan, Italy) supplemented with 50 mg/L calcium. *S. aureus* ATCC-29213 was included for quality control of antimicrobial susceptibility patterns. Cefoxitin disk diffusion test was used to confirm oxacillin resistance (EUCAST, version 4.0, 2014) [23].

Inducible clindamycin resistance was evaluated using the D-zone test as recommended by EUCAST (Version 4.0, 2014) [23].

### Detection of *S. epidermidis me*c*A* gene

The presence of the ***mecA*** gene in *S. epidermidis* strains was investigated by PCR using primers and conditions described by Vannuffel *et al*. [24].

### Characterization of slime-producing ability

The phenotypic characterization of slime-producing ability by culture of the strains on CRA plates, prepared by adding 0.8 g of Congo red (Sigma, Missouri) and 36 g of saccharose (Sigma, Missouri) to 1 L of brain heart infusion agar (Oxoid, Basingstoke, Hampshire, England) as described by Freeman *et al.* [25]. *S. aureus* colonies on CRA were kept under observation for up to 72 h, as described by Arciola *et al*. [26].

### Biofilm assays

The presence and extent of biofilm structures, produced by staphylococci was quantified spectrophotometrically at 570 nm using MtP plates following the indications described by Christensen *et al.,* [27] and as reported previously by this group [28]. In accordance with the original method, strains with OD values < 0.120 were considered as negative, those with ODs >0.120 and <0.240 were regarded as weak biofilm-producers. An OD value > 0.240 was indicative of biofilm-producing bacterial strains. For each isolate the MtP test was repeated in triplicate.

In order to assess glucose- and NaCl-induced biofilm formation, bacteria were grown in 96-well polystyrene microtitre plates in BHI medium or BHI supplemented with 1% glucose or 4% NaCl at 37°C for 24 h. Biofilms were quantified spectrophotometrically as described by O’Neill *et al*. [29].

Biofilm stability against proteinase K, sodium metaperiodate and DNaseI treatment was tested for both species as described previously [29]. Effect of DNaseI on biofilm development was assessed as described by Houston *et al.* [30].

Two-tailed, two sample equal variance Student’s t-tests (Microsoft Excel 2007) were used to determine statistically significant differences in biofilm-forming capacity and biofilm stability.

### PCR-method for the detection of MSCRAMM and biofilm genes

The presence of *ica*A and *ica*D genes was detected by PCR using primers and conditions described by O’Neill *et al*., Rohde *et al.*, Arciola *et al*. and de Silva *et al*. [26,29,31,32].

The genes encoding the autolysins/adhesins responsible for primary attachment to abiotic surfaces (*atlA/ atlE* and *aae*), the components causing intercellular aggregation (*bap*, *sas*G, *bhp*, *aap*) and microbial surface components recognizing adhesive matrix molecules (MSCRAMMs) *bbp, cna, fib, fnbA, fnbB, clfA, clfB, fbe, ebpS, eno, sdrF* and *embp*, were amplified by PCR using the primers and conditions summarized in (Additional file 6: Table S3) [33-45].

### Expression of MSCRAMM and biofilm genes

Expression of MSCRAMM and biofilm genes was assessed by Reverse transcription (RT)-PCR for all staphylococcal strains.

RNA was purified from cultures grown at 37°C in BHI medium supplemented with 1% glucose (strains producing glucose-induced biofilms) or 4% NaCl (strains producing NaCl-induced biofilms) to an A_600_ of 2.0. Total bacterial RNA was isolated using the High Pure RNA Isolation Kit (Roche, Mannheim, Germany) according to the manufacturer's instructions. After purification, contaminating DNA was removed with DNase I recombinant RNase-free (10 U/40 μg of total bacterial RNA) at 37°C for 20 min. The RNA was then repurified using RNeasy Mini columns (Qiagen, Inc.). The amount of RNA recovered was determined spectrophotometrically, and the absence of DNA was verified by PCR using primers described in the Multilocus sequence typing (MLST) database (http://www.mlst.net) corresponding to the constitutively expressed phosphate acetyltransferase gene (*pta*) for *S. aureus* and the carbamate kinase gene (*arcC*) for *S. epidermidis*. Samples were then stored at −80°C.

RT-PCR was performed using the Transcriptor First Strand cDNA synthesis kit (Roche, Mannheim, Germany) and the cDNA was directly used as a template to amplify selected MSCRAMM and biofilm genes using the same primers and PCR conditions mentioned earlier (Additional file 6: Table S3). PCR products were separated by electrophoresis in a 1.2% agarose gel and visualized with ethidium bromide under UV light**.**

### Detection of *S. aureus* capsular and leukotoxin genes

PCR amplification of *S. aureus cap5* (to assess for genes encoding capsular type 5), and *cap8* (to assess for genes encoding capsular type 8), was performed using primers and conditions described by Sau *et al.*, [46].

Detection of the Panton-Valentine leukocidin (PVL) and LukE/LukD and LukM leukotoxin genes was performed for all *S. aureus* isolates by PCR as previously reported [47,48] (Additional file 6: Table S3).

### RAPD

RAPD analysis was performed on *S. aureus* strains using three separate primers, AP-PCR1 (5′-ggTTgggTgAgAATTgcAcg) AP-PCR7 (5′-gTggATgcgA) and AP-PCR ERIC-2 (5′-AAgTAAgTgAcTGGGGTgAgCg), with PCR conditions described by Van Belkum *et al.* [49].

For *S. epidermidis* strains RAPD analysis was performed using primers AP-PCR ERIC-2 and AP-PCR7.

The RAPD patterns were considered to be different when the profiles differed by at least one band.

### *S. aureus* MLST and eBURST analysis

MLST was performed for all *S. aureus* isolates according to the protocol described on the *S. aureus* MLST website (http://saureus.mlst.net). The allele types and the resulting sequence types were determined by submitting the allelic profile of representative alleles to the *S. aureus* MLST database via the Internet. Sequence types were clustered into groups by eBURST analysis (v3.0 software) with a stringent group definition with six of seven loci to determine the clonal relationship of the isolates.

### *S. aureus* agr typing

For all *S. aureus* the *agr* locus was typed by a multiplex PCR as previously described [50].

## Additional files

Additional file 1: Table S1. Genetic and phenotypic characteristics of 13 S. aureus strains studied. Additional file 2: Table S2. Genetic and phenotypic characteristics of 7 *S. epidermidis* strains studied. Additional file 3: Figure S1. Comparison of *ica* operon expression in *S. aureus* and *S. epidermidis* grown in BHI medium or in BHI supplemented with 4% NaCl or 1% glucose (Glu). Additional file 4: Figure S2. RAPD fingerprinting of the 13 *S. aureus* strains studied. RAPD profiles generated by primer AP-PCR1 (A), primer AP-PCR7 (B) and primer AP-PCR ERIC-2 (C). The DNA molecular weight marker XIV (Roche, Mannheim, Germany) is in lanes 1 and 15. Additional file 5: Figure S3. RAPD fingerprinting of the 7 *S. epidermidis* strains studied. RAPD profiles generated by primer AP-PCR7 (A) and primer AP-PCR ERIC-2 (B). The DNA molecular weight marker XIV (Roche, Mannheim, Germany) is in lanes 1 and 9. Additional file 6: Table S3. Primers used for PCR-based detection of staphylococcal factors involved in the pathogenesis of foreign-body associated infections.

## Abbreviations

MSCRAMMs: Microbial surface components recognizing adhesive matrix molecules
(PIA): Extracellular polysaccharide promoting intercellular adhesion
PNAG: Polymeric N-acetyl-glucosamine
CRA: Congo red agar
MtP: Polystyrene microtiter plates
BHI: Brain heart infusion
OD: Optical density
RAPD: Randomly amplified polymorphic DNA
PCR: Polymerase chain reaction
PJI: Prosthetic joint infections
MRSA: Methicillin-resistant *S. aureus*
MSSA: Methicillin-susceptible *S. aureus*
MS: Methicillin-sensible
aap: Accumulation-associated protein
MIC: Minimum inhibitory concentration
EUCAST: European committee for antimicrobial susceptibility testing
RT: Reverse transcription
MLST: Multi locus sequencing typing
PVL: Panton-Valentine leukocidin
CC: Clonal complex
