# Supplementary material for: Phenotypic and genotypic characterization of Staphylococci causing breast peri-implant infections in oncologic patients
Source: BMC Microbiol. 2015 Feb 10;15(1):26. doi: 10.1186/s12866-015-0368-x (PMC4328704; doi:10.1186/s12866-015-0368-x)
Supplement: Additional file 1: Table S1. — Genetic and phenotypic characteristics of 13 S. aureus strains studied. [file 12866_2015_368_MOESM1_ESM.pdf]

Table S1. Genetic and phenotypic characteristics of 13 *S. aureus* strains studied.

Strains	MSCRAMM and biofilm genes studied														capsular type	Biofilm formation*			Antibiotype**										
	ebpS	eno	AtlA	bap	sasG	cna	bbp	fnbA	fnbB	clfA	clfB	fib	icaA	ica D		BHI	BHI glucose	BHI NaCl	CIP	SXT	DA	E	TE	RD	LZD	VA	OXA	CN	DPC
**SA1**	+	+	+	-	+	+	-	+	-	+	-	-	+	+	8	w	m	s	S	S	S	S	S	S	S	S	S	S	S
**SA4**	+	+	+	-	-	+	-	+	-	+	+	+	+	+	8	m	s	w	S	S	S	R	S	S	S	S	S	S	S
**SA7**	+	+	+	-	-	+	-	+	-	+	-	-	+	+	8	m	s	w	S	S	S	S	S	S	S	S	S	S	S
**SA 11**	-	+	+	-	+	+	-	+	+	+	+	+	+	+	8	m	s	w	S	S	S	S	S	S	S	S	S	S	S
**SA18**	+	+	+	-	+	+	-	+	-	+	+	+	+	+	5	m	s	w	S	S	S	R	S	S	S	S	S	S	S
**SA21**	-	+	+	-	+	+	-	+	+	+	+	+	+	+	5	m	s	w	S	S	S	S	S	S	S	S	S	S	S
**SA24**	+	+	+	-	+	+	-	+	-	+	-	+	+	+	5	w	m	s	S	S	S	S	S	S	S	S	S	S	S
**SA 25**	-	+	+	-	+	+	-	+	-	+	+	+	+	+	5	w	m	s	S	S	S	S	S	S	S	S	S	S	S
**SA35**	+	+	+	-	+	+	-	+	-	+	+	+	+	+	8	m	s	w	S	S	S	R	S	S	S	S	S	S	S
**SA 36**	+	+	+	-	+	+	-	+	-	+	-	+	+	+	5	m	s	w	S	S	S	S	S	S	S	S	S	S	S
**SA 38**	+	+	+	-	-	+	-	+	-	+	-	-	+	+	8	m	s	w	S	S	S	S	S	S	S	S	S	S	S
**SA 418**	+	+	+	-	+	+	-	+	-	+	+	-	+	+	5	w	m	s	S	S	S	S	S	S	S	S	S	S	S
**SA 420**	+	+	+	-	+	+	-	+	-	+	+	+	+	+	5	w	m	s	R	S	S	R	R	R	R	R	R	R	R

* w: weakly adherent, m: moderately adherent; s: strongly adherent

** CIP: ciprofloxacin; SXT: cotrimoxazole; DA: clindamicina; E: erythromycin; TE: tetracycline; RD: rifampicin; LZD: linezolid; VA: vancomycin; OXA: oxacillin; CN: gentamicin; DPC: daptomycin
