# Supplementary material for: Phenotypic and genotypic characterization of Staphylococci causing breast peri-implant infections in oncologic patients
Source: BMC Microbiol. 2015 Feb 10;15(1):26. doi: 10.1186/s12866-015-0368-x (PMC4328704; doi:10.1186/s12866-015-0368-x)
Supplement: Additional file 2: Table S2. — Genetic and phenotypic characteristics of 7 S. epidermidis strains studied. [file 12866_2015_368_MOESM2_ESM.pdf]

Table S2. Genetic and phenotypic characteristics of 7 *S. epidermidis* strains studied.

Strains	MSCRAMM and biofilm genes studied									Biofilm formation*			Antibiotype**										
	AtlE	aae	aap	bhp	fbe	sdrF	embp	icaA	ica D	BHI	BHI glucose	BHI NaCl	CIP	SXT	DA	E	TE	RD	LZD	VA	OXA	CN	DPC
**SE 5**	+	+	+	-	-	-	+	-	+	m	s	w	S	S	S	R	S	S	S	S	R	S	S
**SE 9**	+	+	+	-	+	+	+	-	-	m	s	w	S	I	S	S	S	S	S	S	S	S	S
**SE 17**	+	+	+	-	+	+	+	-	-	m	s	w	S	S	S	R	S	S	S	S	R	S	S
**SE 20**	+	+	+	-	+	-	+	-	-	m	s	w	S	S	S	S	S	S	S	S	S	R	S
**SE 29**	+	+	+	-	+	-	+	+	+	w	m	s	S	S	S	R	S	S	S	S	S	S	S
**SE 32**	+	+	+	-	+	+	+	-	-	m	s	w	S	S	S	R	S	S	S	S	S	S	S
**SE 40**	+	+	-	-	+	+	+	-	-	m	s	w	S	S	S	S	S	S	S	S	S	R	S

* w: weakly adherent, m: moderately adherent; s: strongly adherent
**CIP: ciprofloxacin; SXT: cotrimoxazole; DA: clindamicina; E: erythromycin; TE: tetracycline; RD: rifampicin; LZD: linezolid; VA: vancomycin; OXA: oxacillin; CN: gentamicin; DPC: daptomycin
