# Supplementary material for: Phenotypic and genotypic characterization of Staphylococci causing breast peri-implant infections in oncologic patients
Source: BMC Microbiol. 2015 Feb 10;15(1):26. doi: 10.1186/s12866-015-0368-x (PMC4328704; doi:10.1186/s12866-015-0368-x)

Figure S1. Comparison of *ica*A expression in 13 *S. aureus* and *2 S. epidermidis* grown in BHI medium or in BHI supplemented with 4% NaCl or 1% glucose (Glu).

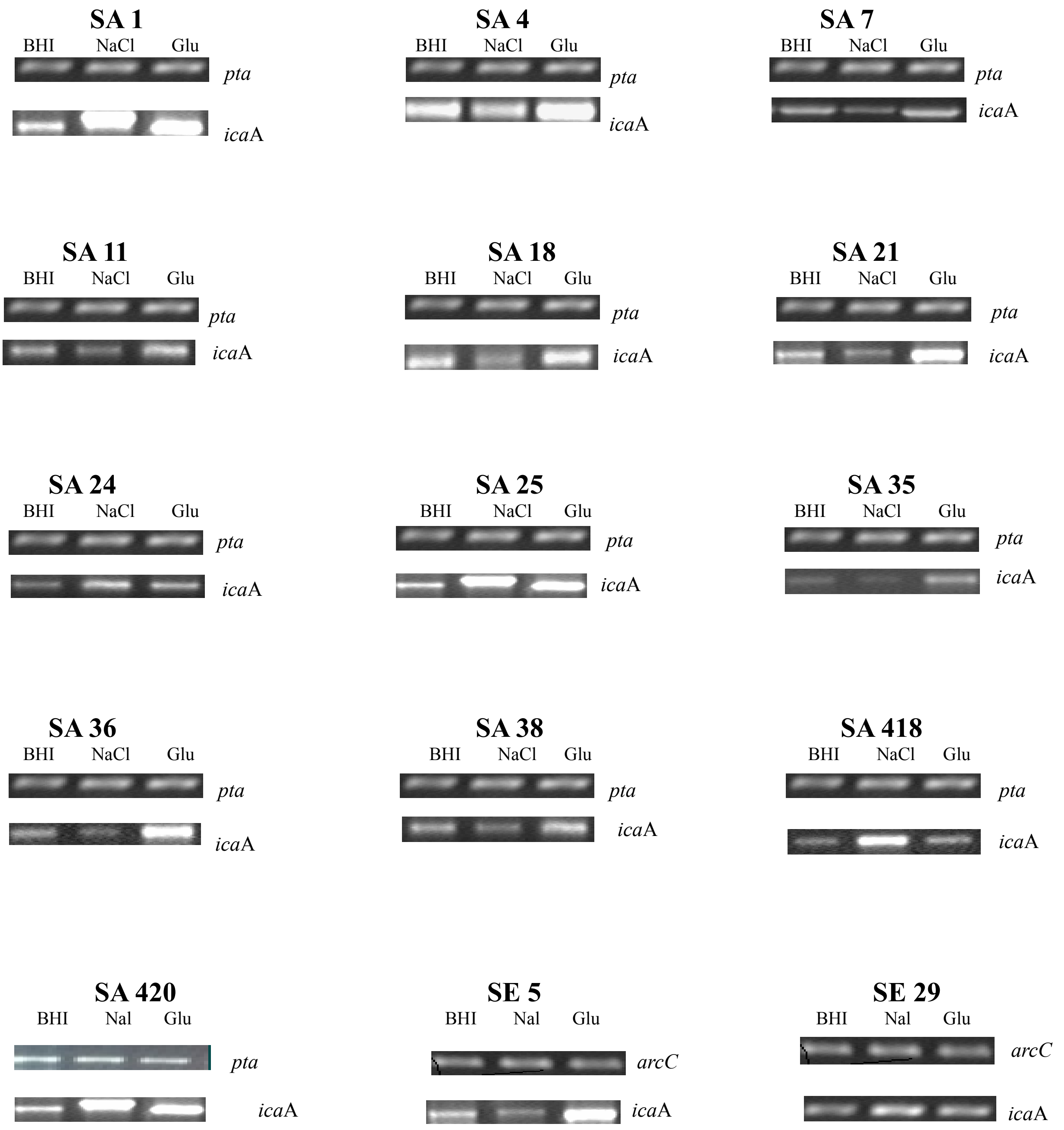

Supplement: Additional file 3: Figure S1. — Comparison of ica operon expression in S. aureus and S. epidermidis grown in BHI medium or in BHI supplemented with 4% NaCl or 1% glucose (Glu). [file 12866_2015_368_MOESM3_ESM.pdf]
