# Supplementary figures and images for: Phenotypic and genotypic characterization of Staphylococci causing breast peri-implant infections in oncologic patients
Source: BMC Microbiol. 2015 Feb 10;15(1):26. doi: 10.1186/s12866-015-0368-x (PMC4328704; doi:10.1186/s12866-015-0368-x)

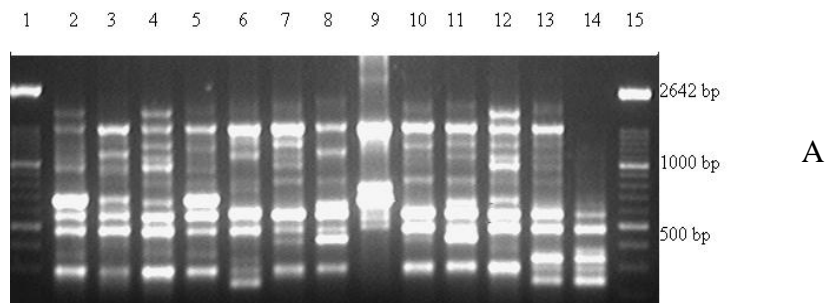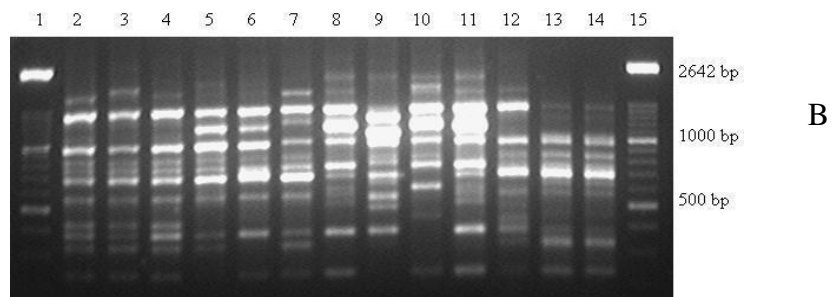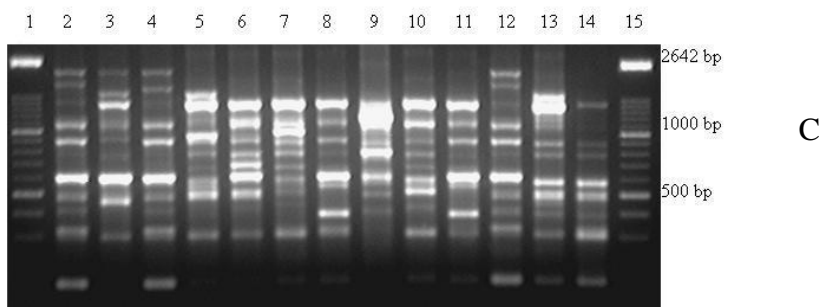

**Figure S2. RAPD fingerprinting of the 13 *S. aureus* strains studied.**

Supplement: Additional file 4: Figure S2. — RAPD fingerprinting of the 13 S. aureus strains studied. RAPD profiles generated by primer AP-PCR1 (A), primer AP-PCR7 (B) and primer AP-PCR ERIC-2 (C). The DNA molecular weight marker XIV (Roche, Mannheim, Germany) is in lanes 1 and 15. [file 12866_2015_368_MOESM4_ESM.pdf]

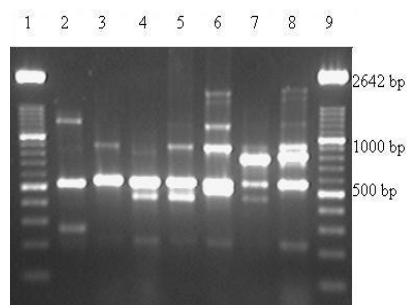

A

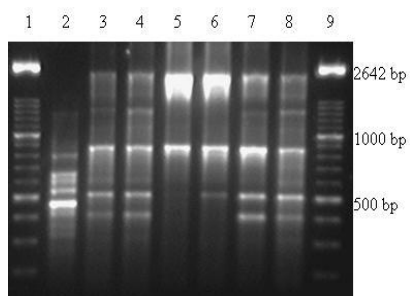

B

**Figure S3. RAPD fingerprinting of the 7 *S. epidermidis* strains studied.**

Supplement: Additional file 5: Figure S3. — RAPD fingerprinting of the 7 S. epidermidis strains studied. RAPD profiles generated by primer AP-PCR7 (A) and primer AP-PCR ERIC-2 (B). The DNA molecular weight marker XIV (Roche, Mannheim, Germany) is in lanes 1 and 9. [file 12866_2015_368_MOESM5_ESM.pdf]
