# Supplementary material for: Phenotypic and genotypic characterization of Staphylococci causing breast peri-implant infections in oncologic patients
Source: BMC Microbiol. 2015 Feb 10;15(1):26. doi: 10.1186/s12866-015-0368-x (PMC4328704; doi:10.1186/s12866-015-0368-x)
Supplement: Additional file 6: Table S3. — Primers used for PCR-based detection of staphylococcal factors involved in the pathogenesis of foreign-body associated infections. [file 12866_2015_368_MOESM6_ESM.pdf]

Table S3. Primers used for PCR-based detection of staphylococcal factors involved in the pathogenesis of foreign-body associated infections

Gene	Putative function of encoded protein	Primer	PCR conditions [Ref.]
S. aureus			
fnbA	Fibronectin adhesin	Forward 5'-CAT AAA TTG GGA GCA GCA TCA-3'	[42]
		Reverse 5'-ATC AGC AGC TGA ATT CCC ATT-3'	
fnbB	Fibronectin adhesin	Forward 5'-GTA ACA GCT AAT GGT CGA ATT GAT ACT-3'	[40]
		Reverse 5'-CAA GTT CGA TAG GAG TAC TAT GTT C-3'	
clfA	Fibrinogen adhesin	Forward 5'-ATT GGC GTG GCT TCA GTG CT-3'	[40]
		Reverse 5'-GTT TCT TCT GTA GTT GCA TTT G-3'	
clfB	Fibrinogen adhesin	Forward 5'-TCG GTT GGA ATA ATG AGA ATG T-3'	[43]
		Reverse 5'-TTT CGC TCT TAT CTC CTG TTT C-3'	
cna	Collagen adhesion	Forward 5'-AAA GCG TTG CCT AGT GGA GA-3'	[41]
		Reverse 5'-AGT GCC TTC CCA AAC CTT TT-3'	
ebpS	Elastin adhesion	Forward 5'-CAT CCA GAA CCA ATC GAA GAC-3'	[40]
		Reverse 5'-AGT TAC ATC ATC ATG TTT ATC TTT TG-3'	
sasG	Adherence to desquamated nasal epithelial cells	Forward 5'-CGA GCT TTT CTA ACC TTA GGT GTC-3'	[37]
		Reverse 5'-ACC ACA GGG TGT AGA AGC TAA ATC-3'	
icaA	N-acetyl-D-glucosamine synthase involved in PIA synthesis	Forward 5'-CGC ACT CAA TCA AGG CAT TA-3'	[29]
		Reverse 5'-CCA GCA AGT GTC TGA CTT CG-3'	
icaD	Intercellular adhesion proteinD	Forward 5'-ATG GTC AAG CCC AGA CAG AG	[31]
		Reverse 5'-TTG CTT TAA ACA TTG AAA ATA CT-3'	
bap	Involved in biofilm formation in *S. aureus* bovine mastitis isolates	Forward 5'-CCC TAT ATC GAA GGT GTA GAA TTG CAC-3'	[36]
		Reverse 5'-GCT GTT GAA GTT AAT ACT GTA CCT GC-3'	
eno	Binds laminin	Forward 5'-ACG TGC AGC AGC TGA CT-3'	[40]
		Reverse 5'-CAA CAG CAT CTT CAG TAC CTT C-3'	

atlA	Autolysin	Forward 5'-CCG TTA CCT GTT TCT AAT AGG-3'	[32]
		Reverse 5'-CAG TTA GCA AGA TTG CTC AAG-3'	
bbp	Binds bone sialoprotein	Forward 5'-AAC TAC ATC TAG TAC TCA ACA ACA G-3'	[40]
		Reverse 5'-ATG TGC TTG AAT AAC ACC ATC ATC T-3'	
fib	Binds fibrinogen	Forward 5'-CTA CAA CTA CAA TTG CGT CAA CAG-3'	[40]
		Reverse 5'-GCT CTT GTA AGA CCA TTT TCT TCA C-3'	
lukM	Toxin	Forward 5'-TGG ATG TTA CCT ATG CAA CCT AC-3'	[48]
		Reverse 5'-GTT CGT TTC CAT ATA ATG AAT CAC TAC -3'	
lukDE	Toxin	Forward 5'-TGA AAA AGG TTC AAA GTT GAT ACG AG-3'	[48]
		Reverse 5'-TGT ATT CGA TAG CAA AAG CAG TGC A-3'	
lukS/F-PV	Panton Valentine leukocidin	Forward 5'-ATC ATT AGG TAA AAT GTC TGG ACA TGA TCC A-3'	[47]
		Reverse 5'-GCA TCA AGT GTA TTG GAT AGC AAA AGC-3'	
cps5	capsular polysaccharide type 5	Forward 5'-GGTTTGCTGAAAAACCAGTC-3'	[46]
		Reverse 5'-CCTCATATGCTCCTACATTT-3'	
cps8	capsular polysaccharide type 8	Forward5'-GCGCTACAAACATTAAGCAT-3'	[46]
		Reverse5'-TTCTTAGCCTGCTGGCATC-3'	
agr$_{Sa}$	gene regulator	5'-ATGCACATGGTGCACATGC-3'	[50]
agr-1$_{Sa}$	gene regulator	5'-GTCACAAGTACTATAAGCTGCGAT-3'	[50]
agr-2$_{Sa}$	gene regulator	5'-TATTACTAATTTGAAAAGTGCCATAGC-3'	[50]
agr-3$_{Sa}$	gene regulator	5'-GTAATGTAATAGCTTGTATAATAATACCCAG-3'	[50]
agr-4$_{Sa}$	gene regulator	5'CGATAATGCCGTAATACCCG-3'	[50]
S. epidermidis			
fbe	Fibrinogen adhesin	Forward 5'-TAA ACA CCG ACG ATA ATA ACC AAA-3'	[42]
		Reverse 5'-GGT CTA GCC TTA TTT TCA TAT TCA-3'	
embp	Fibronectin adhesin	Forward 5'-AGC GGT ACA AAT GTC AAT ATC-3'	[45]
		Reverse 5'-AGA AGT GCT CTA GCA TCA TCC-3'	

atlE	Promotes binding to polystyrol; vitronectin adhesin	Forward 5'-CAA CTG CTC AAC CGA GAA CA-3'	[34]
		Reverse 5'-CAT CGT TTT CAG CGC TAT CA-3'	
aae	Autolysin	Forward 5'-AAC AAA TTG ATA AAG CAA CG-3'	[35]
		Reverse 5'-GTT GTC TTT CCT TTA GTG TC-3'	
aap	Intercellular adhesin	Forward 5'-TCA CTA AAC AAC CTG TTG ACG AA-3'	[39]
		Reverse 5'-AAT TGA TTT TTA TTA TCT GTT GAA TGC-3'	
icaA	N-acetyl-D-glucosamine synthase involved in PIA synthesis	Forward 5'-GAC CTC GAA GTC AAT AGA GGT-3'	[26]
		Reverse 5'-CCC AGT ATA ACG TTG GAT ACC-3'	
icaD	Intercellular adhesion proteinD	Forward 5'-ATG GTC AAG CCC AGA CAG AG-3'	[32]
		Reverse 5'-CGT GTT TTC AAC ATT TAA TGC AA-3'	
bhp	Homologue to biofilm associated protein Bap of *S. aureus*	Forward 5'-TGG TAT TAG GAA GCT CTC AG-3'	[38]
		Reverse 5'-ATA CCA GCG TGA CGC AAA TC-3'	
sdrF	Collagen adhesion	Forward 5'-AGC GGC TGA AGA CAA TCA AT-3'	30 sec 95°C, (15 sec 95°C, 15 sec 55°C, 15 sec 60°C) x 30
		Reverse 5'-TCA CTT GTC GTT GGT GCT TC-3'	
